# Antibacterial activity of selected Malaysian honey

**DOI:** 10.1186/1472-6882-13-129

**Published:** 2013-06-10

**Authors:** Mohd Izwan Zainol, Kamaruddin Mohd Yusoff, Mohd Yasim Mohd Yusof

**Affiliations:** 1 Department of Biomedical Science, Faculty of Medicine, University of Malaya, 50603 Kuala Lumpur, Malaysia; 2 Department of Molecular Biology, Faculty of Arts and Science, Canik Basari University, Samsun, Turkey; 3 Department of Medical Microbiology, Faculty of Medicine, University of Malaya, 50603 Kuala Lumpur, Malaysia

**Keywords:** Antibacterial activity, Malaysian honey, Minimum inhibitory concentration (MIC), Minimum bactericidal concentration (MBC), Equivalent phenol concentration (EPC), Non-peroxide activity

## Abstract

**Background:**

Antibacterial activity of honey is mainly dependent on a combination of its peroxide activity and non-peroxide components. This study aims to investigate antibacterial activity of five varieties of Malaysian honey (three monofloral; acacia*, gelam and* pineapple*,* and two polyfloral; *kelulut* and *tualang*) against *Staphylococcus aureus, Bacillus cereus, Escherichia coli*, and *Pseudomonas aeruginosa.*

**Methods:**

Minimum Inhibitory Concentration (MIC) and Minimum Bactericidal Concentration (MBC) were performed for semi-quantitative evaluation. Agar well diffusion assay was used to investigate peroxide and non-peroxide activities of honey.

**Results:**

The results showed that *gelam* honey possessed lowest MIC value against *S. aureus* with 5% (w/v) MIC and MBC of 6.25% (w/v). Highest MIC values were shown by pineapple honey against *E. coli* and *P. aeruginosa* as well as acacia honey against *E. coli* with 25% (w/v) MIC and 50% (w/v) MBC values. Agar inhibition assay showed *kelulut* honey to possess highest total antibacterial activity against *S. aureus* with 26.49 equivalent phenol concentrations (EPC) and non-peroxide activity of 25.74 EPC. Lowest antibacterial activity was observed in acacia honey against *E. coli* with total activity of 7.85 EPC and non-peroxide activity of 7.59 EPC. There were no significant differences (p > 0.05) between the total antibacterial activities and non-peroxide activities of Malaysian honey. The intraspecific correlation between MIC and EPC of *E. coli* (r = -0.8559) was high while that between MIC and EPC of *P. aeruginosa* was observed to be moderate (r = -0.6469). *S. aureus* recorded a smaller correlation towards the opposite direction (r = 0.5045). In contrast, *B.cereus* showed a very low intraspecific correlation between MIC and EPC (r = -0.1482).

**Conclusions:**

Malaysian honey, namely *gelam*, *kelulut* and *tualang,* have high antibacterial potency derived from total and non-peroxide activities, which implies that both peroxide and other constituents are mutually important as contributing factors to the antibacterial property of honey.

## Background

Two important enzymes known to contribute to the major biological activities of honey are bee-origin glucose oxidase and floral-origin catalase [1]. These enzymes are crucial in determining the level of peroxide activity in honey which underlies numerous biological functions, including antibacterial potency. A high amount of active glucose oxidase will hydrolyze glucose to produce hydrogen peroxide (H_2_O_2_) resulting in oxidative stress which is beneficial in controlling bacterial colonization. In contrast, a high catalase level together with high antioxidant capacity will destroy H_2_O_2_ and serve as the principle guarding mechanism in order to conserve the nutritional value of honey.

However, in undiluted honey, glucose oxidase is inactive [1]. Therefore, with assistance from various antioxidant constituents, H_2_O_2_ level in undiluted honey is said to be minimized. Very high osmotic pressures coupled with high acidity are the two main factors contributing to the antibacterial properties of honey at this stage [2,3]. When honey is diluted to certain extents, glucose oxidase will be activated and start to utilize glucose to produce H_2_O_2_. At this point, the antibacterial activity of honey will gradually shift from osmotic- and pH-dependent to peroxide-dependent. Some types of honey, such as manuka honey from New Zealand, possess high non-peroxide antibacterial activity that can retain antibacterial potency even after removing the peroxide component from diluted honey [3,4]. This is known as active non-peroxide honey which contains numerous non-peroxide constituents that support antibacterial actions. These include phenolic compounds, flavonoids, antibacterial peptides, methylglyoxal, methyl syringate, antibiotic-like derivatives and other components present in trace amounts [5,6]. Not all types of honey exhibit non-peroxide activity. Some are strongly peroxide-dependent and possess very low antibacterial action when treated with catalase to remove peroxide activity [3,4,7].

To date, various studies have been conducted to investigate the antibacterial properties of honey from different parts of the world [4,7-13]. White *et al.*[1] proposed that inhibines, the antibiotic system in honey, were linearly related to H_2_O_2_ accumulation while Allen *et al.*[4] subsequently described non-peroxide antibacterial activity in selected New Zealand honey. Limited studies have been done on Malaysian honey. In brief, Tan *et al.*[11] compared the antibacterial activity of *tualang* honey with manuka honey from New Zealand while Aljadi *et al.*[14] isolated and identified phenolic acids from *gelam* and coconut honey and tested them for their antibacterial activity. Tumin *et al.*[15] studied antibacterial activities of five local honey varieties, namely, *tualang*, *hutan*, *gelang*, *pucuk daun* and Ee Feng Gu against various pathogenic bacteria strains.

The emergence of antibiotic-resistant bacteria has drawn major attention among healthcare and medical practitioners ever since the discovery of penicillin in the late 1920s [16]. Several types of bacteria have been reported to develop new strains which survive antibiotics, including *staphylococci*, *enterococci* and *mycobacteria*. The situation became worse when multidrug resistant strains were discovered during the first decade of the 21st century [16]. At the same time, healthcare-associated infections became a serious problem in hospitals [17]. Furthermore, the pharmaceutical industry failed to develop new antimicrobial agents to face the new threat due to the high cost of drug research. In Malaysia, *Pseudomonas aeruginosa*, *Staphylococcus aureus*, and *Escherichia coli* were recorded as among the most common healthcare-associated pathogens isolated from patients [18]. These bacteria may also develop multidrug resistant strains in various circumstances as reviewed by Bereket *et al*[17]. To combat this serious situation, honey seems to be a promising alternative. It has been reported to be effective against a wide range of clinically isolated multi-resistant bacteria, such as, Vancomycin-resistant *Enterococci* (VRE) and multi-resistant *Pseudomonas aeruginosa*[19,20]. The fact that honey resistance has never been reported nor any toxicity or side effects, low cost of maintenance, and local availability confer valuable advantages to using honey as an alternative antimicrobial therapy [6].

This study investigated the antibacterial potential of five Malaysian honey varieties against four bacteria species, namely, *Staphylococcus aureus*, *Escherichia coli* and *Pseudomonas aeruginosa,* which are widely known to cause multidrug-resistant healthcare-associated infections, as well as *Bacillus cereus* to represent the spore-forming bacteria species known to cause food poisoning [17,18,21,22].

## Methods

### Honey samples

Honey samples were obtained from local apiarists and stored in the dark at room temperature. The identification was performed by the bee hunters based on their geographical hunting area and floral availability at the location of bee hives (foraging radius). These were supported by organoleptic confirmation of the honey. The five types of honey used were: (i) acacia; honey derived from a plant widely used in the forest plantation industry from Sarawak state of Malaysia known as tropical acacia species or *Acacia mangium*, (ii) *gelam*; honey derived from mangrove swamp in Johore state known as *Melaleuca cajuputi* powell, (iii) *kelulut*; this type of honey is harvested by a stingless bee species, *Trigona* spp., and derived from multifloral foraging activity of bees, (iv) pineapple; a monofloral variety derived from pineapple flowers, *Ananas comosus*, and (v) *tualang*; a wild polyfloral honey produced by *Apis dorsata* located on one of the tallest tropical rainforest trees from species *Koompassia excelsa.* To ascertain the reproducibility and reliability of our study, the standard commercially available medical grade honey derived from manuka tree was included (*Comvita* Wound Care UMF 18+, New Zealand).

### Bacteria

Cultures of bacteria were supplied by Microbiology Laboratory, University of Malaya Medical Centre (UMMC). *Bacillus* species was obtained from Molecular Bacteriology Laboratory, Department of Biomedical Science, University of Malaya. All bacteria were of standard strains (ATCC, US) comprising two Gram-positive bacteria; *Staphylococcus aureus* (ATCC 25923) and *Bacillus cereus* (ATCC 11778) and two Gram-negative bacteria; *Escherichia coli* (ATCC 25922) and *Pseudomonas aeruginosa* (ATCC 27853). Bacteria supplied were reconstituted into Trypticase Soy broth, TS (Difco,US) and incubated at 37°C. After 24 hours, they were sub-cultured on Mueller Hinton Agar, MH (Lab M, UK) and incubated again at 37°C for another 24 hours before being processed for long storage at -80° in cryogenic vials (Nalgene, US) containing brain Heart infusion broth, BHI (Difco, US) and 15% glycerol (R & M Chemicals, UK).

Working bacterial culture was prepared by inoculating a loopful of primary culture from -20° storage into universal bottle containing 10 ml of TS broth. The inoculum was incubated at 37°C for 24 hours before proceeding to subsequent assay.

### Reagents and chemicals

All reagents and chemicals were of analytical grade or better. The diluent used in this study was sterile deionized distilled water (Merck Milipore, US), unless stated otherwise. Artificial honey was prepared by dissolving 40.5% fructose (sigma, US), 33.5% glucose (sigma, US), 7.5% maltose (sigma, US) and 1.5% sucrose (sigma, US) in a final volume of 100 ml sterile deionized distilled water [20].

### Minimum inhibitory concentration (MIC) and minimum bactericidal concentration (MBC)

The MIC test was adapted from Patton *et al.*[23] and Tan *et al.*[11] with slight modifications. Working bacteria culture was prepared as previously described, adjusted to be equal to 0.5 McFarland standard (1 × 10^8^ cfu/ml) and further diluted by mixing 1 part of adjusted culture with 199 parts of TS broth to meet 5 × 10^5^ cfu/ml. Volumes of 10 ml TS broth was pipetted into five sterile screw-capped test tubes and labeled accordingly. Another empty tube served as the first tube of honey stock solution where it was used to prepare 50% (w/v) honey solution by weighing 5 g honey sample, made up to 10 ml with TS broth, well mixed and filtered through 0.2 μm filters (Sartorius AG, Germany). A Two-fold serial dilution was prepared using all five pre-filled tubes together with four extra tubes containing honey dilutions of 5, 10, 15 and 20% (w/v). All tubes were vortex (Digisystem Laboratory Instruments Inc., Taiwan) until uniformly mixed. A volume of 190 μl of each honey dilution was aseptically transferred into 96 well flat-bottom microtitre plates (Nunc, Denmark) in eight replicates per dilution. The first two wells of every honey dilution served as dilution sterility controls (added with another 10 μl of respective honey dilution) and six others were the test wells in which 10 μl bacteria culture was mixed. Row number 11 and 12 were reserved for batch sterility and growth controls. Volume of 200 μl TS broth was used as assay sterility control in all wells of row 11 while 10 μl bacteria culture in 190 μl TS broth served as the assay growth control in all wells of row 12. Plates were incubated in a shaker incubator (Stuart, UK) at 120 rpm, 37°C for 24 hours. The absorbance of the wells was read at 590 nm using microtitre plate reader (Bio-rad, US) after incubation. The percentages of inhibition of bacteria growth were calculated by using the following formula:

$$1-\left(\mathrm{Absorbance}\;\mathrm{of}\;\mathrm{test}\;\mathrm{well}-\mathrm{Absorbance}\;\mathrm{of}\;\mathrm{corresponding}\;\mathrm{control}\;\mathrm{well}\right)/\left(\mathrm{Absorbance}\;\mathrm{of}\;\mathrm{assay}\;\mathrm{growth}\;\mathrm{control}-\mathrm{Absorbance}\;\mathrm{of}\;\mathrm{sterility}\;\mathrm{control}\right)\times 100.$$

Standards were prepared according to well-established two-fold dilution method comprising phenol (sigma, US), ampicillin (10 mg/L; sigma, US), ciprofloxacin hydrochloride (5 mg/L; Bayer Healthcare Pharmaceuticals, US) and tetracycline (30 mg/L; sigma, US) [24,25].

MBC test was performed after MIC assay via streak plate method. Each honey dilution with no bacteria growth from the MIC test was determined. For each honey concentration with no bacteria growth, two wells of the corresponding honey dilution were randomly selected and one loopful bacteria suspension was transferred from each well onto two separate MH agars. It was spread evenly and incubated at 37°C for 24 hours. MBC were determined by the minimum concentration that allowed less than 1% of bacterial growth.

### Agar well diffusion assay

The assay method was adapted from Allen *et al.*[4] with slight modifications. Volumes of 150 ml nutrient agar, NA (Difco, US) was prepared according to manufacturer’s instructions. It was allowed to cool after autoclaving (at high pressure, 121°C for 10 minutes) and standing at 50°C before being seeded with 100 μl of 24 hours bacteria culture (prepared to meet absorbance of 0.5 measured at 540 nm using TS broth as diluent and blank). After uniform swirling, the agar was poured into large square bioassay dishes (245 × 245 × 25 mm; Nunc, Denmark). Solidified plates were stored overnight at 4°C upside down to be used the following day. Wells were cut into the agar using a sterile cork borer with 8 mm diameter.

Honey samples were freshly prepared for each assay and filter-sterilized with 0.2 μm filters. Twenty five percent (w/v) honey in deionized distilled water was prepared for the total activity test and 25% (w/v) honey in catalase solution (5 mg/mL, Fluka, Germany) for the non-peroxide activity test. Aliquots of 100 μl well-mixed honey samples were transferred randomly into each corresponding well in quadruplicate. Sterile deionized distilled water and catalase solution were used as a blank in duplicates for every plate. Phenol standards 1% (w/v) to 10% (v/v) were prepared and transferred in the same manner as the samples application. Phenol standards can be used up to one month when stored at 4°C. Plates were then incubated at 37°C for 24 hours. Ampicillin (10 μg), ciprofloxacin (5 μg) and tetracycline (30 μg) were included to ascertain the reproducibility and reliability of the assay and the bacterial resistant profiles.

The diameter of zones of inhibition of the wells was measured using digital vernier calipers (Mitotoyo, Japan) by measuring them in at least 2 directions perpendicular to each other (90°). The measurements were performed before all the samples and standards were re-identified to avoid bias. The mean of diameters of inhibition zone for each well and honey sample was calculated and squared. A standard curve was plotted of phenol concentration (%, v/v) against the mean of square diameter of inhibition zone. The best-fit linear line was drawn and the equation generated was used to calculate honey antibacterial activity from the readings obtained (Excel, Microsoft Corporation, US) expressed as Equivalent Phenol Concentration, EPC (%, w/v).

### Catalase effectiveness test (removal of H_2_O_2_ confirmation)

*Gelam* and *tualang* honey were chosen to test the effectiveness of catalase using the method by Allen *et al.*[4]. In brief, 6 tubes of test solution were prepared and labeled: tube 1 (25% (w/v) honey solution, 45 mmol/L H_2_O_2_ and 5 mg/mL catalase solution), tube 2 (25% (w/v) honey solution and 5 mg/mL catalase solution), tube 3 (45 mmol/L H_2_O_2_ and 5 mg/mL catalase solution), tube 4 (25% (w/v) honey solution and 45 mmol/L H_2_O_2_), tube 5 (25% (w/v) honey solution and tube 6 (45 mmol/L H_2_O_2_). Solutions then were tested in the same way as agar well diffusion assays on the same plate, and the diameters of clear zones (mm) were recorded.

### Data analysis

Student’s *t*-test with two-tailed distribution (Excel, Microsoft Corporation, US) was used to compare total and non-peroxide activities of Malaysian honey. Pearson’s correlation (Excel, Microsoft Corporation, US) was performed to evaluate the association between diameter of inhibition zones expressed in EPC and minimum inhibitory concentration (MIC) expressed in percentage (%, w/v).

## Results

**Figure 1:**
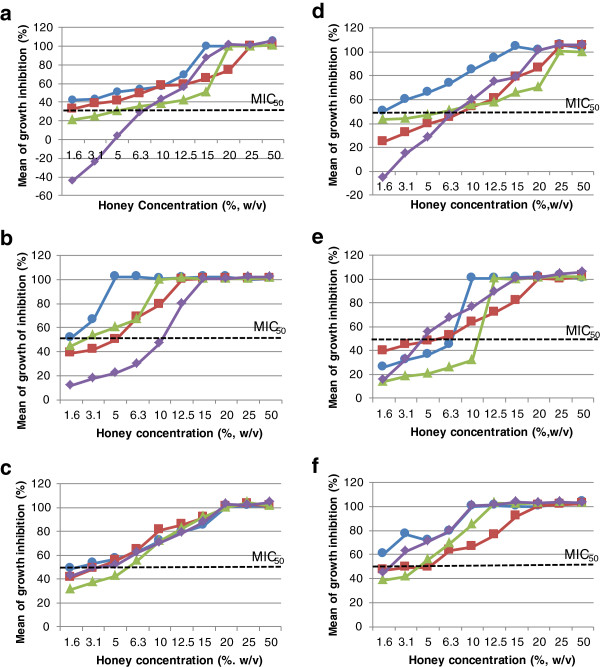
**Percentage of bacteria growth inhibition by Malaysian honey. a**) acacia honey; **b**) *gelam*; **c**) *kelulut*; **d**) pineapple; **e**) *tualang*; **f**) Manuka (*Comvita* 18+) against *S. aureus* (circle, blue), *E. coli* (square, red*), P. aeruginosa* (triangle, green) and *B. cereus* (diamon, purple).

The MIC and MBC values of Malaysian honey against the four tested bacteria are shown in Table 1. G*elam* honey was recorded as the most potent honey against *S. aureus*, in which a dilution of only 5% (w/v) was required to inhibit and kill them at 6.25% (w/v). The highest concentration required was 15% (w/v) to simultaneously inhibit and kill *B. cereus*. Interestingly, *kelulut* honey demonstrated constant MIC and MBC results at 20% (w/v) for all tests. *Tualang* honey closely resembled New Zealand Manuka honey (*Comvita* UMF 18+). The only difference between them was detected against *B. cereus* whereby *tualang* exerted slightly higher MIC and MBC values. Acacia honey had the lowest antibacterial potency to all bacteria tested except for *E. coli,* against which pineapple honey was least effective. Overall, the bactericidal activities of Malaysian honey were recorded to be one reading higher than their inhibitory effects with the exception of *kelulut* honey. Results of artificial honey showed that all bacteria were inhibited at 50% (w/v) concentration and no bactericidal effect was recorded. Phenol standards were found to be effectively inhibit bacterial growth at very low concentration, as low as 0.5 to 1% (w/v), while MBC was as low as 1 to 2% (w/v) only. Figure 1 displays the details of bacterial growth response against Malaysian honeys in MIC test. All bacteria showed dose–response activity in various degrees. *Kelulut* honey appeared most consistent in inhibiting bacterial growth regardless of their species. This was proven by the growth inhibition curve (Figure 1c) showing stable or similar increments in inhibition percentage of bacterial growth with little difference between bacteria species compared to other types of honey. In general, bacterial growth inhibition started at a level below MIC_50_ and increased gradually with honey concentration until it reached 100%. At low concentrations of acacia honey (1.6 and 3.1%, w/v) and pineapple honey (1.6% w/v), negative values were recorded (Figures 1a &1d) against B. cereus.

**Table 1:** Minimum Inhibitory Concentration (MIC) & Minimum Bactericidal Concentration (MBC) of Malaysian honey

**Honey samples**	**MIC/MBC (%, w/v)**							
	***S. aureus***		***E. coli***		***P. aeruginosa***		***B. cereus***	
	**MIC**	**MBC**	**MIC**	**MBC**	**MIC**	**MBC**	**MIC**	**MBC**
Acacia	15	25	25	50	20	50	20	25
*Gelam*	5	6.25	12.5	15	10	12.5	15	15
*Kelulut*	20	20	20	20	20	20	20	20
Pineapple	15	25	25	50	25	50	20	25
*Tualang*	10	15	20	25	12.5	20	15	20
Manuka (*Comvita* 18+)	10	15	20	25	12.5	20	10	12.5
Artificial honey (std)	50	>50^a^	50	>50^a^	50	>50^a^	50	>50^a^
Phenol solution (std)	0.5	2	1	2	0.5	1	1	1
Ampicillin (std)	0.25	0.25	4	4	>128^a^	NT	16	16
Ciprofloxacin (std)	0. 5	1	0.125	0.25	0.25	0.25	0.125	0.25
Tetracycline (std)	0.125	0.125	2	4	64	128	0.063	0.063

MIC was defined as 99% bacteriostatic effects; MBC was defined as 99% of bacterial killing effect, ^a^; the highest concentration tested. NT; Not Tested, n = 6.

**Figure 2:**
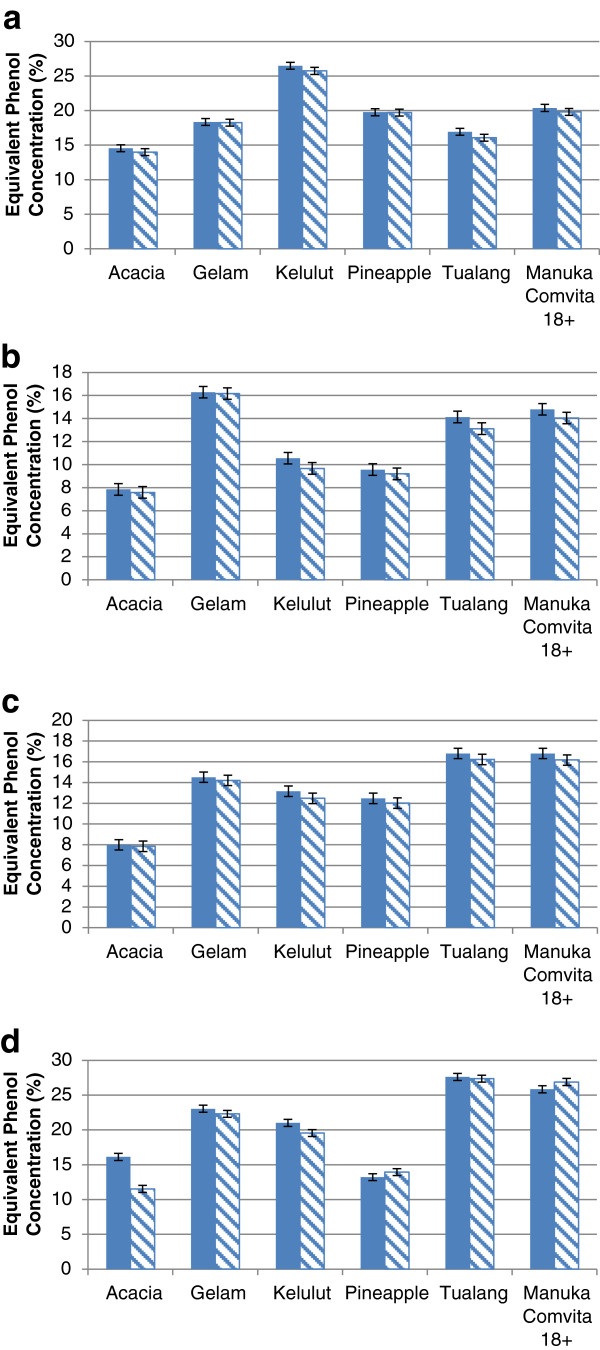
**Total and non-peroxide antibacterial activity of Malaysian honey. a**) *S. aureus*; **b**) *E. coli*; **c**) *P. aeruginosa*; **d**) *B. cereus*. Filled; total activity, diagonal; non-peroxide activity. Student’s *t*-test confirmed that there were no significant differences between total and non-peroxide antibacterial activities for all honey tested (p > 0.05).

Table 2 shows measurements of EPC of Malaysian honey against four tested bacteria in terms of total activity and H_2_O_2_ exclusion activity. Four sets of the data showed very high activities, i.e., over 20 EPC. These were *kelulut* honey against *S. aureus* and the other three, namely, *gelam*, *tualang* and manuka (+18) honey against *B. cereus*. Three sets of data showed low antibacterial activities (below 10 EPC) - acacia honey against *E. coli* and *P. aeruginosa*, and pineapple honey against *E. coli*. *Kelulut* honey against *E. coli* showed total activity over 10 EPC but non-peroxide activity below 10 EPC. Comparisons between total and non-peroxide activities of Malaysian honey are shown in Figure 2. The differences between the two sets of data of the same honey were apparently not far apart except for acacia honey against *B. cereus* (Figure 2d). Furthermore, as all the differences were not statistically significant (p > 0.05), based on student’s *t*-test with 2-tailed distribution to compare total and non-peroxide activities, the data were not presented.

**Table 2:** Agar well diffusion assay for antibacterial activities of Malaysian honey against tested bacteria

**Honey samples**	**Antibacterial activity, Equivalent Phenol Concentration (EPC)**							
	***S. aureus***		***E. coli***		***P. aeruginosa***		***B. cereus***	
	**Total**	**Non-peroxide**	**Total**	**Non-peroxide**	**Total**	**Non-peroxide**	**Total**	**Non-peroxide**
Acacia	14.56	13.99	7.85	7.59	8.00	785	16.12	11.52
*Gelam*	18.35	18.25	16.28	16.17	14.51	14.20	23.04	22.31
*Kelulut*	26.49	25.74	10.56	9.67	13.16	12.48	21.01	19.55
Pineapple	19.76	19.71	9.57	9.20	12.48	12.01	13.21	13.94
*Tualang*	16.94	16.08	14.13	13.12	16.80	16.22	27.61	27.35
Manuka (*Comvita* +18)	20.38	19.81	14.80	14.04	16.80	16.17	25.84	26.88

Equivalent phenol concentration (EPC) was calculated in undiluted honey. Square of mean diameter of clear zone were multiplied by dilution factor and density of Malaysian honey, 1.3 w/v to obtain 100% honey concentration. n = 4 [4,15].

**Figure 3:**
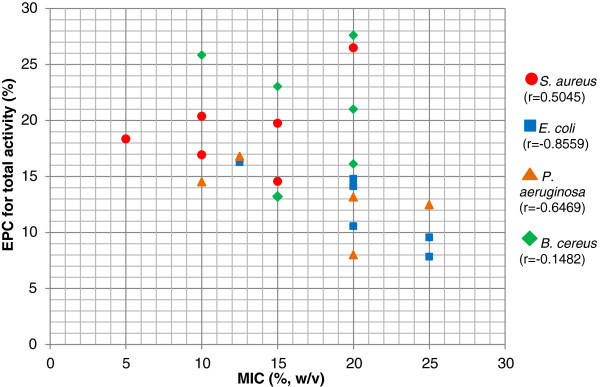
**Scatter plot employed to find association between MIC and EPC of Malaysian honey against four bacteria tested.***S. aureus* (circle), *E. coli* (square*), P. aeruginosa* (triangle) and *B. cereus* (diamond). Pearson’s correlation coefficient, r (parentheses) was calculated to demonstrate intraspecific bacterial association between MIC and EPC. There was no correlation between MIC and EPC of entire bacteria population obtained (r = -0.2848).

The scatter plot (Figure 3) demonstrates a pattern of antibacterial effect for each bacteria species tested. Whilst correlations (r) between MIC and EPC of *P. aeruginosa* were observed to be moderate (r = -0.6469), *S. aureus* also recorded moderate correlations, but towards the opposite direction (r = 0.5045). *E. coli* however, showed a high r value (r = -0.8559) indicating a strong correlation between MIC value and EPC. In contrast, there was no correlation at all between MIC value and EPC for *B. cereus* (r = -0.1483). All data were distributed randomly across the plotted area suggesting no direct correlation between the four tested bacteria population and the five Malaysian honey varieties (r = -0.2848).

## Discussions

The antibacterial activity of honey has been assayed using various methods across the globe with special attention devoted to agar diffusion assay and minimum inhibitory concentration (MIC) coupled with minimum bactericidal concentration (MBC). In this study, MIC was performed using 96-well microtitre plate and data were collected by means of a spectrophotometric endpoints evaluation. This method was chosen based on a number of reasons, including high sensitivity, reproducibility and repeatability, less time consumption, reduced cost, fewer amounts of sample and reagent required, and most importantly, less subjectivity as it does not involve human observations with the naked eye. Authors Patton *et al.*[23], Brudzynski *et al.*[26] and Sherlock *et al.*[13] used T_24_-T_0_ different time comparison to measure the antibacterial effect of honey. In our preliminary test, T_24_-T_0_ different time comparisons showed a critical problem of inconsistency in the results recorded. In honey sterility control wells, the final readings (T_24_) deviated from initial readings (T_0_) detected. Some honey gave increased spectrophotometric readings while others showed otherwise even though at high honey concentration, this was expected to be constant due to the absence of bacterial growth. We suspected this could be due to volatile compounds present in honey [27-29]. At initial time (T_0_), these compounds were still in a complex mixture within the honey solution, hence, were measured as part of the sample. After 24 hours, under incubation temperature of 37°C, some volatile compounds could have evaporated, thus, affecting the measurements recorded. A significant reduction in the spectrophotometric reading led to false evaluation of bacterial growth. The degree of reduction of spectrophotometric readings was suggested to be dependent on the amount of volatile compounds in honey. As a complex mixture of different molecules and compounds, the other chemical constituents of honey might also affect its absorbance, including minerals, peptides, amino acids and alkaloids which can produce major interference [28]. Therefore, this method of measurement was avoided and single endpoints (T_24_) method of measurement was chosen.

MIC is defined as the lowest concentration of honey that prevents at least 99% of bacterial growth while MBC is defined as the lowest concentration of honey required to kill at least 99% of the bacteria. Equal bacteriostatic and bactericidal effects of *kelulut* honey deviated from all other readings whereby all of them exhibited higher MBC value than MIC. The findings suggest that *kelulut* honey has different effects on the tested bacteria regardless of their species and survival abilities. This could be due to the presence of different organic antibacterial factors contributed by stingless bee (*Trigona* spp) rather than the usual honey bee (*Apis* spp), as well as the floral origin of the nectar [7,27]. Previous findings reported by Tan *et al.*[11] stated that manuka honey (*Korde*l’s, UMF 10+) exhibits higher antibacterial activity than *tualang* honey against *S. aureus*, *E. coli* and *P. aeruginosa*. In comparison, our study obtained a lower MIC for *tualang* honey against three bacteria, *S. aureus*, *E. coli* and *P. aeruginosa* probably due to different batch of honey and technical variations. Nonetheless, the pattern of antibacterial response of this particular honey was in agreement, i.e., S*. aureus* was found to be the most susceptible bacteria, followed by *P. aeruginosa* and *E. coli*.

MIC_50_ was defined as the concentration of honey required to inhibit bacterial growth by 50%. We included the MIC_50_ value in our data presentation (Figure 1) to give a clearer picture of bacterial growth inhibition patterns. Stimulation of *B. cereus* growth, denoted by negative values (Figures 1a & d), in low concentrations of acacia and pineapple honey might be due to the concentration of glucose which is sufficient to support *B. cereus* growth but not concentrated enough to inhibit them by osmotic pressure. At the lowest honey concentration tested, most honey inhibited more than 20% of bacterial growth with a few exceptions. *B. cereus* was the most unaffected bacteria when treated with low honey concentration, except for *kelulut* and manuka honey. Despite the adaptive ability of *Bacillus* species which are capable of withstanding alteration of their surrounding environment by forming spores, *B. cereus* were still inhibited and eventually killed by all types of honey tested [22]. However, no further test was done to ascertain whether *B. cereus* were totally killed or were sporulating to withstand the antibacterial effects of honey.

Agar diffusion assay for antibacterial activity test is usually performed in three different ways - well/cup diffusion, disk diffusion or agar dilution. The method of choice usually depends on the nature of antibacterial agents to be tested and the kinetic properties of molecules inside. In our study, we decided to use well diffusion assay because honey is a complex solution consisting of different sizes of chemicals and compounds [28]. The use of disk could lead to the exclusion of large molecules which are not properly absorbed by the paper disks and may contribute to inaccurate results. Agar well exercise allows direct contact of honey components and bacteria immediately after application. The diffusion mechanism may also represent *in-vivo* conditions when honey is applied on infected wounds, and therefore, may provide information about the kinetic system of honey application. This method was performed to evaluate the antibacterial activity of Malaysian honey at fixed concentration (qualitatively) compared to semi-quantitative evaluation by MIC/MBC tests. Specifically, *S. aureus* was most susceptible to *kelulut* honey, *E. coli* was most affected by *gelam* honey, *P. aeruginosa* was equally susceptible to *tualang* and manuka (+18) honey and *B. cereus* was highly vulnerable to *tualang* honey. Our findings also recorded that some Malaysian honey have higher antibacterial activity compared to well-known manuka (+18) honey as proven by *kelulut* honey against *S. aureus* (Figure 2a) and *gelam* honey against *E. coli* (Figure 2b).

In this study, H_2_O_2_ was removed from the honey solution to measure the antibacterial effect of honey without the presence of peroxide molecules [4]. Student’s *t*-test with 2-tailed distribution was used to compare total and non-peroxide activities of Malaysian honey. The data were not presented because all of the differences were not statistically significant (p > 0.05). From these calculations, Malaysian honey was shown to have high non-peroxide activity. The antibacterial activity was not affected significantly by the absence of H_2_O_2_. They were just slightly reduced to some extent (Figure 2) indicating that H_2_O_2_ is still one of the major components of honey’s antibacterial system. Some of the readings from agar diffusion assay generated interesting information compared to MIC/MBC values. For example, *kelulut* honey exerted high MIC/MBC values (20%, w/v) which theoretically means poor antibacterial effect, but gave large zones of inhibition on agar diffusion assay, especially against *S. aureus,* indicating high antibacterial activity. This contradicting result between the two assays might be due to the properties of their chemical constituents. At high honey concentration, particularly concentrations above MIC value, they easily diffuse throughout the agar and inhibit bacterial growth in a large area. The variation in chemical composition might possibly be due to the unique property of *kelulut* as mentioned earlier. Further analysis on the chemical composition of antibacterial compounds is required to elucidate this. Contradicting results were also detected in the MIC/MBC assays against EPC measurement for *B. cereus*. High values of MIC/MBC data (Table 1) were recorded for this particular bacteria indicating poor antibacterial effect while agar diffusion assay showed high EPC value (Table 2), especially for *gelam*, *kelulut*, *tualang* and manuka (+18). This was the reason why we found no association at all between MIC and EPC of *B. cereus* (Figure 3). A possible explanation might be the adaptive ability of this species, as discussed earlier, which caused the bacteria to be highly affected at a particular level of honey concentration while remaining unaffected at low concentrations.

Our study emphasized that even though honey has high antibacterial potency against some bacteria species, it was not conclusive that they were both quantitatively and qualitatively excellent. In theory, low MIC value should give high EPC value since both are expected to have a high antibacterial potency. As such, the scatter plot (Figure 3) should illustrate a negative correlation between the two. From our data, *S. aureus* had deviated from this hypothesis with opposite results while *B. cereus* was totally in disagreement. This might due to *kelulut* honey which was considered an outlier. Exclusion of *kelulut*’s data resulted in a negative correlation with r = -0.7780 for *S. aureus* (data not shown) which was supports our hypothesis. Apparently, *kelulut* honey is unique and should be tested and analysed separately from the blossom honey. Our findings concurred with the latest antibiotic resistance issue, i.e., the serious therapeutic challenge presented by Gram-negative bacteria, *P. aeruginosa* and *E. coli*, due to its bacterial adaptive mechanism against current available antibiotics [16]. This situation suggests that Gram-negative bacteria are less susceptible to available antibiotics compared to Gram-positive bacteria, which is consistent with our data as shown in Tables 1 and 2.

Honey sample for our study only involved one batch representing each type of Malaysian honey. A larger sample size should be tested and analysed in order to obtain a better picture about their correlation. According to Molan PC [3], a small number of samples does not represent a particular source of honey as a whole. Therefore, this present study was considered more likely to be a preliminary screening of Malaysian honey for their antibacterial potency. Our study used standard laboratory strains because there are very limited studies reporting on the five types of Malaysian honey of interest (acacia, *gelam*, *kelulut*, pineapple and *tualang*) against these bacteria species. *S. aureus* was included as it is widely used as the standard Gram-positive strain of preliminary assay with *E.coli* representing the Gram-negative strain [4,7,23,25,30]. *P. aeruginosa* represented a prominent healthcare-associated pathogen and *B. cereus* was chosen to represent spore-forming species which might also became clinically important particularly in food poisoning [16,21]. To assess the antibacterial potency of these five Malaysian honey against antibiotic-resistant strains, further investigations should be conducted against antibiotic resistant and clinically isolated strains. The effect of these honey varieties on biofilm of bacteria should also be carried out since the present study only testes their effect against planktonic bacteria. Reproducibility and repeatability of the test were verified using commercially available *Comvita* +18 UMF manuka honey against standard strains, *S. aureus* (ATCC 25923). Allen *et al.*[4] stated that +18 UMF means that the honey contains at least 18% (w/v) phenol equivalents of non-peroxide activity. This study proved to be reproducible when *Comvita* +18 Manuka Honey was assayed resulting in 18.38 UMF, sd ± 0.14%. Artificial honey was used to demonstrate the osmotic effect of honey against bacteria preferably to exclude the osmotic factors of natural honey. MIC and diameters of inhibition zones for all antibiotics were reproduced for susceptible strains of all bacteria tested as determined by Clinical Laboratory Standard Institute (CLSI) (data not shown [24,31-33]). The effectiveness of catalase was assayed to affirm that the catalase added was working well in removing all H_2_O_2_ molecules and its activity was not affected by other components (Table 3).

**Table 3:** **Well diffusion assays of catalase test against *S. aureus *(ATCC 25923). The diameter of clear zones obtained in mm (±s.d.), n = 6**

**Solutions**	**Without adding catalase (mm)**		**With addition of catalase (mm)**	
	***Gelam***	***Tualang***	***Gelam***	***Tualang***
25% (w/v) honey solution + 45 mmol/L H_2_O_2_	25.57 (±0.79)	29.27 (±0.77)	15.50 (±0.4)	16.51 (±0.19)
25% (w/v) honey solution	15.52 (±0.13)	16.34 (±0.19)	15.47 (±0.27)	16.26 (±0.11)
45 mmol/L H_2_O_2_	34.68 (±0.16)		No detectable activity	

## Conclusion

The antibacterial potencies of Malaysian honey were generally comparable with well-known New Zealand manuka honey, with close resemblance by *tualang* honey. Agar diffusion assay proved that all Malaysian honey possess high non-peroxide antibacterial activity. Malaysian honey, namely *gelam*, *kelulut* and *tualang* honey have high antibacterial potency of total and non-peroxide activities implying that peroxide and other constituents are mutually important as contributing factors to the antibacterial system of honey. The correlations between MIC and EPC value of Malaysian honey were proven to be dependent on bacteria species and honey origin. The spore-forming bacteria, *B. cereus*, were found to be affected differently by Malaysian honey as compared to other bacteria species. *Kelulut* honey has quantitatively poor but qualitatively excellent antibacterial potency. Gram-positive bacteria proved to be more susceptible to Malaysian honey compared to Gram-negative bacteria species.

## Competing interests

The authors declare that they have no competing interests.

## Authors’ contributions

MIZ planned, carried out the experiments, involved in data interpretation, and prepared the manuscript including revisions. MYMY design and planned the experiments in microbiological aspect, contributed in data interpretation and manuscript/revision editing. KMY acted as project leader and advisor of this study mainly in biochemical part of honey analysis, contributed in data interpretation and troubleshooting as well as manuscript and revision editing. All authors read and approved the manuscript.

## Pre-publication history

The pre-publication history for this paper can be accessed here:

http://www.biomedcentral.com/1472-6882/13/129/prepub
